# Discovery of an Unusual Fatty Acid Amide from the *ndgR_yo_* Gene Mutant of Marine-Derived *Streptomyces youssoufiensis*

**DOI:** 10.3390/md17010012

**Published:** 2018-12-28

**Authors:** Jing Hou, Jing Liu, Lu Yang, Zengzhi Liu, Huayue Li, Qian Che, Tianjiao Zhu, Dehai Li, Wenli Li

**Affiliations:** 1 Key Laboratory of Marine Drugs, Ministry of Education, School of Medicine and Pharmacy, Ocean University of China, Qingdao 266003, China; 17864275159@163.com (J.H.); liujing900908@163.com (J.L.); xihongshi94@163.com (L.Y.); liuzz1990@outlook.com (Z.L.); cheqian1396@sina.com (Q.C.); zhutj@ouc.edu.cn (T.Z.); dehaili@ouc.edu.cn (D.L.); 2 Laboratory for Marine Drugs and Bioproducts of Qingdao National Laboratory for Marine Science and Technology, Qingdao 266237, China

**Keywords:** *NdgR_yo_*, IclR family regulator, *Streptomyces*, fatty acid amide, genome mining

## Abstract

*NdgR_yo_*, an IclR-like regulator, was selected as the target gene to activate new secondary metabolites in the marine-derived *Streptomyces youssoufiensis* OUC6819. Inactivation of the *ndgR_yo_* gene in *S*. *youssoufiensis* OUC6819 led to the accumulation of a new fatty acid amide (**1**), with an unusual 3-amino-butyl acid as the amine component. Moreover, its parent fatty acid (**2**) was also discovered both in the wild-type and *ΔndgR_yo_* mutant strains, which was for the first time isolated from a natural source. The structures of compounds **1** and **2** were elucidated by combination of LC-MS and NMR spectroscopic analyses. This study demonstrated that the *ndgR_yo_* homologs might serve as a target for new compound activation in *Streptomyces* strains.

## 1. Introduction

Marine *Streptomyces* have evolved unique abilities to adapt to the marine environment, which ensures their survival in extreme habitats (e.g., low temperature, high pressure, and poor nutrients) and provides a variety of novel secondary metabolites [1]. With the increase in the number of sequences deposited in microbial genome databases, an increasing number of secondary metabolite biosynthetic gene clusters have been disclosed; however, the majority of them are silent or barely expressed under ordinary laboratory conditions [2]. Thus, activation of silent gene clusters has become an effective strategy for natural product discovery, attracting more and more scientists to this research field.

Secondary metabolism of *Streptomyces* is controlled by a complicated and elaborate regulatory network [3,4]. The precursors for the biosynthesis of secondary metabolites are usually derived from primary metabolism. Manipulation of the regulators in central metabolism has a far-reaching impact on the production of secondary metabolites [5]. The IclR-like global regulator, *ndgR*, is a representative of this metabolism that is involved in amino acid metabolism and conserved among *Streptomyces* species as well as other actinomycetes [6]. Disruption of *ndgR* in *Streptomyces coelicolor* led to defective differentiation and enhanced actinorhodin production in minimal media containing certain amino acids [7]. In *Streptomyces clavuligerus*, deletion of *areB*, a homolog of *ndgR*, resulted in increased production of clavulanic acid and cephamycin C [8].

**Figure 1:**
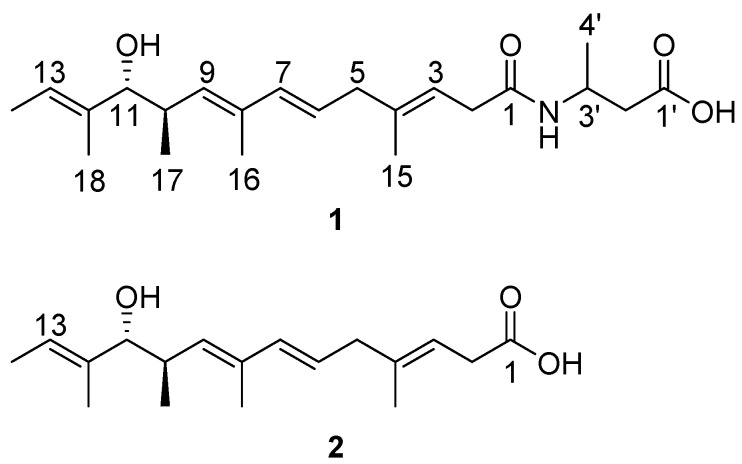
Structures of compounds **1** and **2**.

In our effort to discover novel natural products from the marine-derived *Streptomyces youssoufiensis* OUC6819 by genome mining [9,10], the *ndgR_yo_* gene was selected as a target for compound activation. The disruption of the *ndgR_yo_* gene caused accumulation of a new fatty acid amide (**1**) that shares similar UV spectrum with its parent fatty acid (**2**) present in the wild-type strain (Figure 1). Herein, we describe the isolation, structure elucidation as well as biological evaluation of compounds **1** and **2** from the *ΔndgR_yo_* mutant of *S. youssoufiensis* OUC6819. 

## 2. Results and Discussion

**Figure 2:**
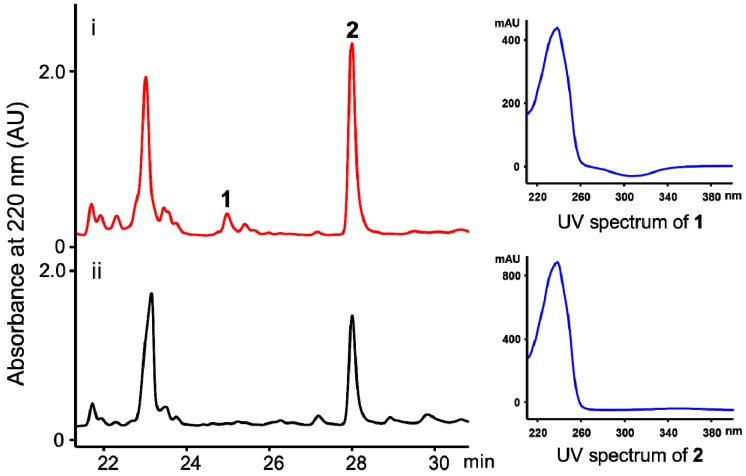
HPLC traces of the fermentation products from the *S. youssoufiensis* OUC6819 strains. (i) The *ΔndgR_yo_* mutant; (ii) the wild-type strain. Compound **1** was newly accumulated in the *ΔndgR_yo_* mutant strain. Compound **2** was produced in both the wild-type and *ΔndgR_yo_* mutant strains, and shares similar UV spectrum with **1**.

The *ndgR_yo_* gene was identified from the *S. youssoufiensis* OUC6819 genome using the local BlastP program. NdgR_yo_ harbors a Helix-Turn-Helix motif at the *N*-terminus, and displays 57% identity to the NdgR from *S. coelicolor* (NP_629686.1). A positive cosmid, pWLI551, was obtained through genome library screening (Table S2). The *ΔndgR_yo_* mutant was obtained using a PCR-targeting strategy, as described in the Section 3.4. The fermentation broths of the wild-type and the *ΔndgR_yo_* mutant strains were extracted with ethyl acetate, and were subsequently subjected to HPLC analysis (Figure 2). The newly accumulated compound **1** in the *ΔndgR_yo_* mutant showed similar UV spectra with that of **2** (Figure 2), indicating they might belong to the same compound class. Large scale fermentation of the *ΔndgR_yo_* mutant resulted in the isolation of compounds **1** and **2**, followed by identification by NMR spectroscopy.

**Figure 3:**
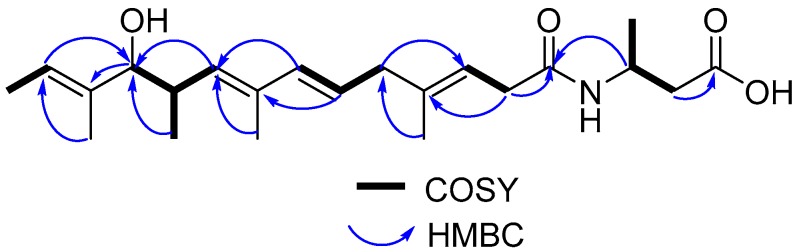
^1^H-^1^H COSY and key HMBC correlations of 1 in CD_3_OD.

**Figure 4:**
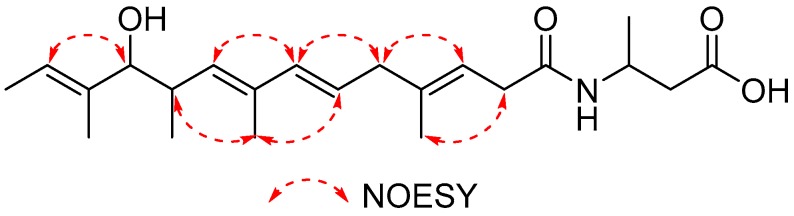
Key NOESY correlations of 1 in CD_3_OD.

Compound **1** was isolated as a yellow oil. The molecular formula of **1** was established as C_22_H_35_NO_4_ (five degrees of unsaturation), as determined by HR-ESIMS data (*m/z* 378.2654 [M + H]^+^, calcd 378.2644) (Figure S1). The structure of **1** was determined from the 1D and 2D NMR (COSY, HSQC, HMBC, and NOESY) data (Figures S2–S7). The ^1^H and HSQC spectra of **1** disclosed six methyl groups (δ_H_ 1.64, 1.66, 1.78, 0.83, 1.63, and 1.21), three methylenes (δ_H_ 2.95, 2.82, 2.54), three methines (δ_H_ 2.71, 3.72, and 4.26), and five olefinic protons (δ_H_ 5.36, 5.57, 6.12, 5.33, and 5.46). The COSY spectrum established five spin systems of H-2 (δ_H_ 2.95)/H-3 (δ_H_ 5.36), H-5 (δ_H_ 2.82)/H-6 (δ_H_ 5.57)/H-7 (δ_H_ 6.12), H-9 (δ_H_ 5.33)/H-10 (δ_H_ 2.71)/H-11 (δ_H_ 3.72)/H-17 (δ_H_ 0.83), H-13 (δ_H_ 5.46)/H-14 (δ_H_ 1.64), and H-2′ (δ_H_ 2.54)/H-3′ (δ_H_ 4.26)/H-4′ (δ_H_ 1.21) (Figure 3). The HMBC correlations from H-13 and H-9 to the hydroxylated carbon C-11 (δ_C_ 82.3), from H-11 to C-12 (δ_C_ 136.7), from H-7 to C-9 (δ_C_ 134.4), from H-6 to C-8 (δ_C_ 133.7), from H-2 to C-4 (δ_C_ 138.4) and a carbonyl carbon C-1 (δ_C_ 172.4), from H-5 to C-3 (δ_C_ 117.3), from H-3′ to C-1, and from H-2′ to C-1′ (δ_C_ 173.2) established the main carbon chain of **1** (Figure 3). The HMBC correlations from the methyl protons H-15 (δ_H_ 1.66) to C-5 (δ_C_ 42.5), H-16 (δ_H_ 1.78) to C-9, H-17 to C-11, and H-18 (δ_H_ 1.63) to C-13 (δ_C_ 121.4), together with the COSY correlations of H-13/H-14 and H-3′/H-4′, confirmed the location of six methyl groups (Figure 3). The ^13^C chemical shifts of C-3′ (δ_C_ 42.2) and C-1, together with the HR-ESIMS data of **1**, revealed the presence of an amide group. Moreover, the configurations of the four double bonds were confirmed to be *E* by NOESY correlations between H-3/H-5, H-5/H-7, H-7/H-9, H-11/H-13, H-2/H-15, H-6/H-16, and H-10/H-16 (Figure 4). The relative configuration between H-10 and H-11 was proposed to be *trans* by the large coupling constant value of 8.4 Hz. Then, the absolute configurations of C-10 and C-11 were determined by comparison of the experimental ECD spectra of **1** and **2** (Figure S15) with calculated ECD spectra of the (2*E*,4*E*,8*E*)-7-methoxy-4,6,8-trimethyldeca-2,4,8-triene moiety reported in the literature [11], which showed high agreement with the 10*R*, 11*R* calculated model. Thus, compound **1** was identified as 3-((3*E*,6*E*,8*E*,10*R*,11*R*,12*E*)-11-hydroxy-4,8,10,12-tetramethyltetradeca-3,6,8,12-tetraenamido) butanoic acid, a new branched-chain fatty acid amide with 3-amino-butyl acid as the amine component. The ^1^H and ^13^C chemical shifts of compound **1** were shown in Table 1.

Compound **2** was isolated as a yellow oil. The molecular formula of **2** was established as C_18_H_28_O_3_ (five degrees of unsaturation), as determined by HR-ESIMS data (*m*/*z* 310.2397 [M + NH_4_]^+^, calcd 310.2382) (Figure S8). The structure of **2** was determined from the 1D and 2D NMR (COSY, HSQC, HMBC, and NOESY) data (Figures S9–S14). According to the ^1^H and ^13^C NMR data (Table 1), compound **2** lacked the 3-amino-butyl acid moiety compared to **1**. Moreover, the NOESY correlations (H-3/H-5, H-5/H-7, H-7/H-9, H-11/H-13, H-2/H-15, H-6/H-16, and H-10/H-16) as well as the experimental ECD spectrum revealed that compound **2** displays the same absolute configurations with **1**. Thus, compound **2** was identified to be the parent fatty acid of **1**, named (3*E*,6*E*,8*E*,10*R*,11*R*,12*E*)-11-hydroxy-4,8,10,12-tetramethyltetradeca-3,6,8,12-tetraenoic acid, which is available from Aurora Fine Chemicals in the United States. Noticeably, this is the first time that compound **2** has been isolated from a natural source.

In the antibacterial activity evaluation of compounds **1** and **2**, neither of them showed obvious inhibitory effects, in the range of concentrations tested, against the multi-drug resistant (MDR) strains, including *Enterococcus faecalis* CCARM 5172, *Enterococcus faecium* CCARM 5203, *Staphylococcus aureus* CCARM 3090, *Escherichia coli* CCARM 1009, and *Salmonella typhimurium* CCARM 8250. Some branched-chain oleic acid derivatives exhibited growth inhibition against MCF-7 and HT-29 cells [12]. Therefore, we also tested the cytotoxicity of compounds **1** and **2** against these two cell lines, but they showed null activity up to the concentration of 50 µM (data not shown).

Fatty acid amides are a class of compounds formed from a fatty acid and an amine that play an important role in intracellular signaling, many of which in nature have ethanolamine as the amine component [13,14,15]. Inactivation of the *ndgR_yo_* gene in *S*. *youssoufiensis* OUC6819 led to the isolation of a new fatty acid amide and (**1**) and its parent branched-chain fatty acid (**2**). The 3-amino-butyl acid moiety in **1** is likely to come from l-glutamate [16]. As the IclR-like global regulator, NdgR, is generally involved in amino acid metabolism [6], we proposed that *ndgR_yo_* in *S. youssoufiensis* OUC6819 might contribute to the generation of the unusual branched-chain fatty acid amide (**1**) with an amino acid as the amine component.

## 3. Materials and Methods 

### 3.1. General Experimental Procedures

1D (^1^H and ^13^C) and 2D (COSY, HSQC, HMBC, and NOESY) NMR spectra were recorded on Bruker Avance III 600 spectrometers at 298 K. The mixing time used for the NOESY spectrum was 142 ms. Chemical shifts were reported with reference to the respective solvent peaks and residual solvent peaks (δ_H_ 3.31 and δ_C_ 49.0 for CD_3_OD). HR-ESIMS data were obtained on a Q-TOF Ultima Global GAA076 MS spectrometer. HPLC was performed on an Agillent 1260 Infinity apparatus equipped with a diode array detector (DAD).

### 3.2. Bacterial Strains and Culture Conditions

*Escherichia coli* DH5α served as the host for general subcloning [17]. *Escherichia coli* ET12567/pUZ8002 was used as the cosmid donor host for *E. coli*-*Streptomyces* intergenic conjugation [18]. *Escherichia coli* BW25113/pIJ790 was used for λRED-mediated PCR-targeting [19]. The *S. youssoufiensis* OUC6819 was isolated from reed rhizosphere soil collected from the mangrove conservation area of Guangdong province, China [9]. *E. coli* strains were routinely cultured in Luria–Bertani (LB) liquid medium at 37 °C, 200 rpm, or LB agar plate at 37 °C. *Streptomyces* strains were grown at 30 °C on R2YE medium for sporulation and ISP4 for conjugation, and were cultured in tryptic soy broth (TSB) medium for genomic DNA preparation. Fermentation medium consists of 1% soluble starch, 2% glucose, 4% corn syrup, 1% yeast extract, 0.3% beef extract, 0.05% MgSO_4_·7H_2_O, 0.05% KH_2_PO_4_, 0.2% CaCO_3_, and 3% sea salt, pH = 7.0.

### 3.3. DNA Isolation and Manipulation

Plasmid extractions and DNA purifications were carried out using standardized commercial kits (OMEGA, Bio-Tek, Guangzhou, China). PCR reactions were carried out using Pfu DNA polymerase (TIANGEN, Beijing, China). Oligonucleotide synthesis and DNA sequencing were performed by TSINGKE company (Qingdao, China).

### 3.4. Gene Inactivation

Positive cosmids harboring the *ndgR_yo_* gene were screened against the genomic library of *S. youssoufiensis* OUC6819 by using PCR with primers listed in Table S1. One cosmid, pWLI551 (Table S2), was obtained and then confirmed by DNA sequencing in TSINGKE company (Qingdao, China). The amplified *aac(3) IV-oriT* resistance cassette from pIJ773 was transformed into *E. coli* BW25113/pIJ790 containing pWLI551 to replace an internal region of the target gene, the PCR primers are listed in Table S3. The mutant cosmid was constructed and introduced into *S. youssoufiensis* OUC6819 by conjugation from *E. coli* ET12567/pUZ8002 according to the reported procedure, using *S. youssoufiensis* OUC6819 ultrasonic fragmented mycelia as acceptors [20]. The desired mutants were selected by the apramycin-resistant and kanamycin sensitive phenotype, and were confirmed by PCR (Figure S16), using the primers listed in Table S4.

### 3.5. Isolation and Purification of the Compounds

The fermentation broth (50 mL) of the *S. youssoufiensis* OUC6819 strains was extracted twice with an equal volume of ethyl acetate, and subsequently subjected to the HPLC analysis. Analytical HPLC was performed on a YMC-Pack ODS-A column (5 μm, 4.6 × 150 mm) developed with a linear gradient from 20% to 100% B/A in 45 min (phase A: H_2_O; phase B: 100% acetonitrile) at the wavelength of 220 nm. The culture broth (15 L) of a scaled-up culture of the *ΔndgR_yo_* mutant was extracted with ethyl acetate and evaporated at room temperature, which was partitioned between 90% methanol and *n*-hexane to remove nonpolar components. Compounds **1** (3 mg) and **2** (10 mg) were obtained by separation of the methanol layer with a linear gradient from 70% to 90% B at a flow rate of 2.0 mL/min using a YMC-Pack ODS-A column (5 μm, 120 Å, 250×10 mm; wavelength 220 nm).

*Compound **1***: Yellow oil; [α] _D_ −8.1 (c 0.1, MeOH); CD (MeOH) λ_max_ (Δε) 202.5 (+5.23), 234.5 (−3.99) nm; ^1^H and ^13^C NMR data, see Table 1; HR-ESIMS *m*/*z* 378.2654 [M + H]^+^ (calcd for C_22_H_36_NO_4_, 378.2644).

*Compound **2***: Yellow oil; [α] _D_ −2.8 (c 0.1, MeOH); CD (MeOH) λ_max_ (Δε) 200.5 (+4.66), 231.5 (−3.51) nm; ^1^H and ^13^C NMR data, see Table 1; HR-ESIMS *m*/*z* 310.2397 [M + NH_4_]^+^ (calcd for C_18_H_32_NO_3_, 310.2382).

### 3.6. Nucleotide Sequence Accession Number

The nucleotide sequence of *ndgR_yo_* in this paper has been deposited in the GenBank database, and the accession number is MH252211.

## 4. Conclusions

A new fatty acid amide (**1**) with an unusual 3-amino-butyl acid as the amine component, together with its parent fatty acid (**2**) were isolated from the *ΔndgR_yo_* mutant strain of *S*. *youssoufiensis* OUC6819. Compounds **1** and **2** displayed neither inhibitory effects against the five MDR bacterial strains, nor cytotoxicity against MCF-7 and HT-29 cancer cell lines. This study demonstrated that the *ndgR_yo_* homologs might serve as a target for activation of structurally novel secondary metabolites in the *Streptomyces* strains.

## Figures and Tables

**Table 1:** ^1^H (600 MHz) and ^13^C (150 MHz) NMR chemical shifts of **1** and **2** in CD_3_OD.

Position	1		2	
	δ_H_ (*J* in HZ)	δ_C_	δ_H_ (*J* in HZ)	δ_C_
1		172.4		174.9
2	2.95 (2H, d, 7.2)	35.0	3.06 (2H, d, 7.2)	33.0
3	5.36 (1H, t, 6.6)	117.3	5.39 (1H, t, 6.6)	116.7
4		138.4		137.7
5	2.82 (2H, d, 7.2)	42.5	2.82 (2H, d, 7.2)	42.5
6	5.57 (1H, dt, 15.6, 7.2)	124.4	5.56 (1H, dt, 15.6, 7.2)	124.4
7	6.12 (1H, d, 15.6)	136.7	6.13 (1H, d, 15.6)	136.7
8		133.7		133.7
9	5.33 (1H, d, 9.0)	134.4	5.33 (1H, d, 9.6)	134.4
10	2.71 (1H, m)	36.1	2.71 (1H, m)	36.1
11	3.72 (1H, d, 8.4)	82.3	3.72 (1H, d, 7.8)	82.3
12		136.7		136.7
13	5.46 (1H, q, 6.6)	121.4	5.47(1H, q, 6.0)	121.4
14	1.64 (3H, d, 6.6)	11.6	1.64 (3H, d, 6.0)	11.7
15	1.66 (3H, s)	15.0	1.66 (3H, s)	15.0
16	1.78 (3H, m)	11.6	1.78 (3H, m)	11.7
17	0.83 (3H, d, 6.6)	16.6	0.83 (3H, d, 7.2)	16.7
18	1.63 (3H, s)	9.7	1.63 (3H, s)	9.7
1′		173.2		
2′	2.54 (1H, dd, 15.6, 6.0) 2.42 (1H, dd, 15.6, 6.0)	40.0		
3′	4.26 (1H, m)	42.2		
4′	1.21 (3H, d, 6.6)	18.8		

## Supplementary Materials

The following are available online at http://www.mdpi.com/1660-3397/17/1/12/s1, Supporting Figures S1–S16 and Tables S1–S4, including HR-ESIMS, NMR and ECD spectra of compounds **1** and **2**, plasmids and primer lists, and biological assay methods.
