# Supplementary material for: Discovery of an Unusual Fatty Acid Amide from the ndgRyo Gene Mutant of Marine-Derived Streptomyces youssoufiensis
Source: Mar Drugs. 2018 Dec 28;17(1):12. doi: 10.3390/md17010012 (PMC6356261; doi:10.3390/md17010012)
Supplement: Supplementary file 1 [file marinedrugs-17-00012-s001.pdf]

Supporting Information

Discovery of an Unusual Fatty Acid Amide from the *ndgR$_{yo}$* Gene Mutant of Marine-Derived *Streptomyces youssoufiensis*

Jing Hou[1], Jing Liu[1], Lu Yang[1], Zengzhi Liu[1], Huayue Li[1,2,*], Qian Che[1,2], Tianjiao Zhu[1,2], Dehai Li[1,2], and Wenli Li[1,2,*]

[1] Key Laboratory of Marine Drugs, Ministry of Education, School of Medicine and Pharmacy, Ocean University of China, Qingdao 266003, China

[2] Laboratory for Marine Drugs and Bioproducts of Qingdao National Laboratory for Marine Science and Technology, Qingdao 266237, China

Table of Contents

Figure S1 The HR-ESIMS spectrum of compound **1**

Figure S2 ^{1}H NMR spectrum of compound **1** in CD_3OD (600 MHz)

Figure S3 ^{13}C NMR spectrum of compound **1** in CD_3OD (600 MHz)

Figure S4 COSY spectrum of compound **1** in CD_3OD (600 MHz)

Figure S5 HSQC spectrum of compound **1** in CD_3OD (600 MHz)

Figure S6 HMBC spectrum of compound **1** in CD_3OD (600 MHz)

Figure S7 NOESY spectrum of compound **1** in CD_3OD (600 MHz)

Figure S8 The HR-ESIMS spectrum of compound **2**

Figure S9 ^{1}H NMR spectrum of compound **2** in CD_3OD (600 MHz)

Figure S10 ^{13}C NMR spectrum of compound **2** in CD_3OD (600 MHz)

Figure S11 COSY spectrum of compound **2** in CD_3OD (600 MHz)

Figure S12 HSQC spectrum of compound **2** in CD_3OD (600 MHz)

Figure S13 HMBC spectrum of compound **2** in CD_3OD (600 MHz)

Figure S14 NOESY spectrum of compound **2** in CD_3OD (600 MHz)

Figure S15 Experimental ECD spectra of compounds **1** and **2**.

Figure S16 Inactivation of $ndgR_{yo}$ (A): Construction of the $\Delta ndgR_{yo}$ gene inactivation mutant. (B): PCR confirmation of the double-crossover mutant.

Table S1 The primer pairs used for cosmid library screening.

Table S2 Bacteria and plasmids used in this study.

Table S3 The primer pairs used for PCR-targeted mutagenesis[a].

Table S4 The primer pairs used for PCR confirmation of the mutant.

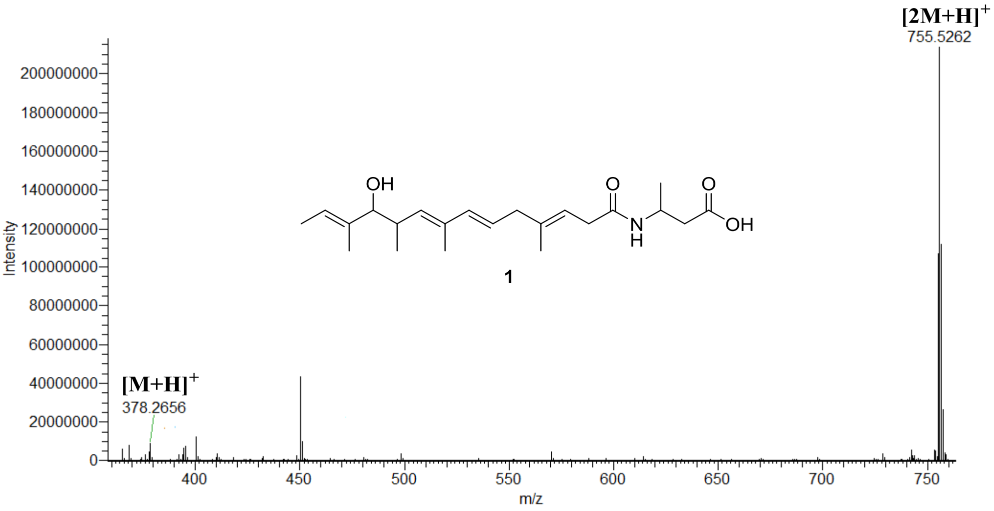

Figure S1. HR-ESIMS spectrum of compound **1**

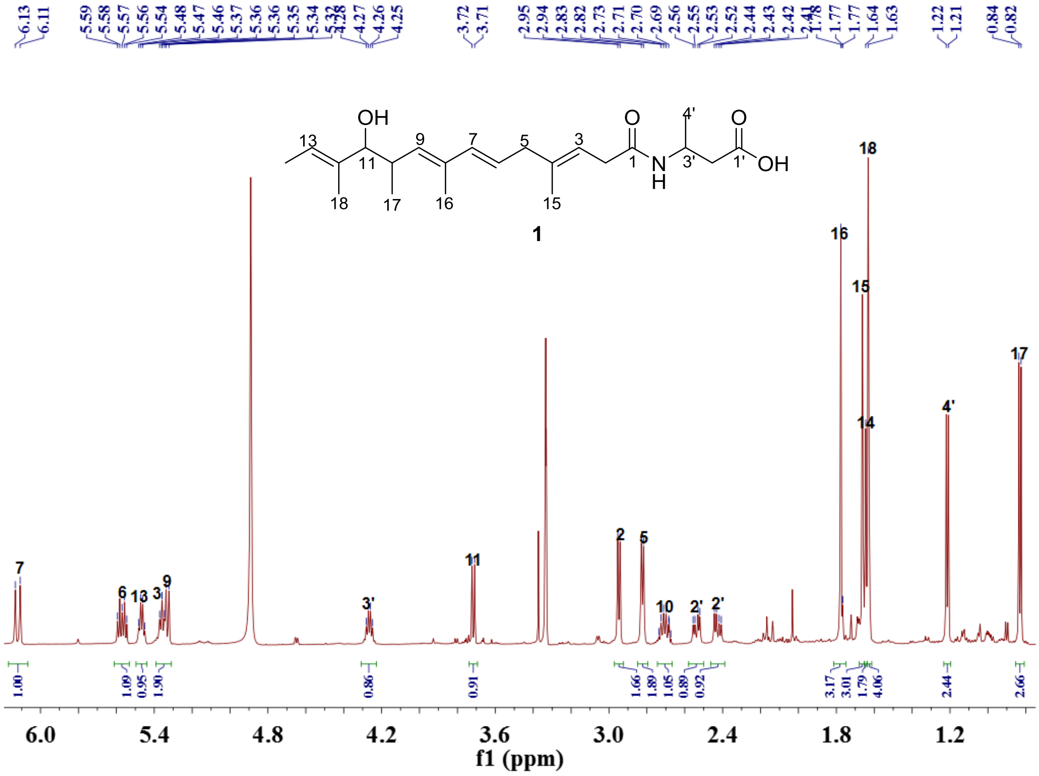

Figure S2. ^{1}H NMR spectrum of compound **1** in CD_3OD (600 MHz).

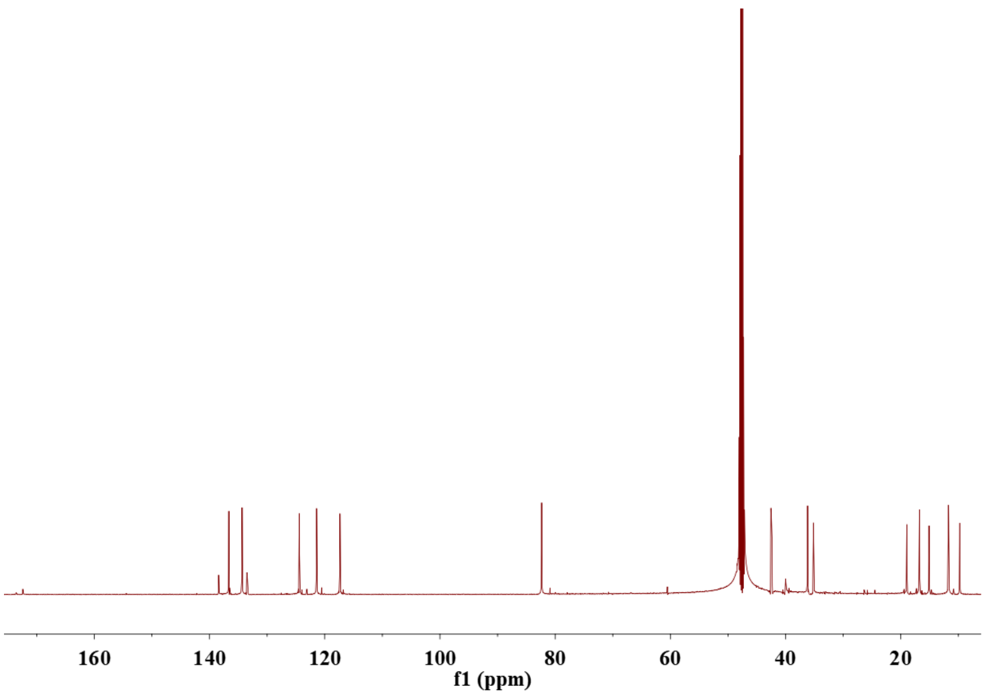

Figure S3. ^{13}C NMR spectrum of compound **1** in CD_3OD (600 MHz).

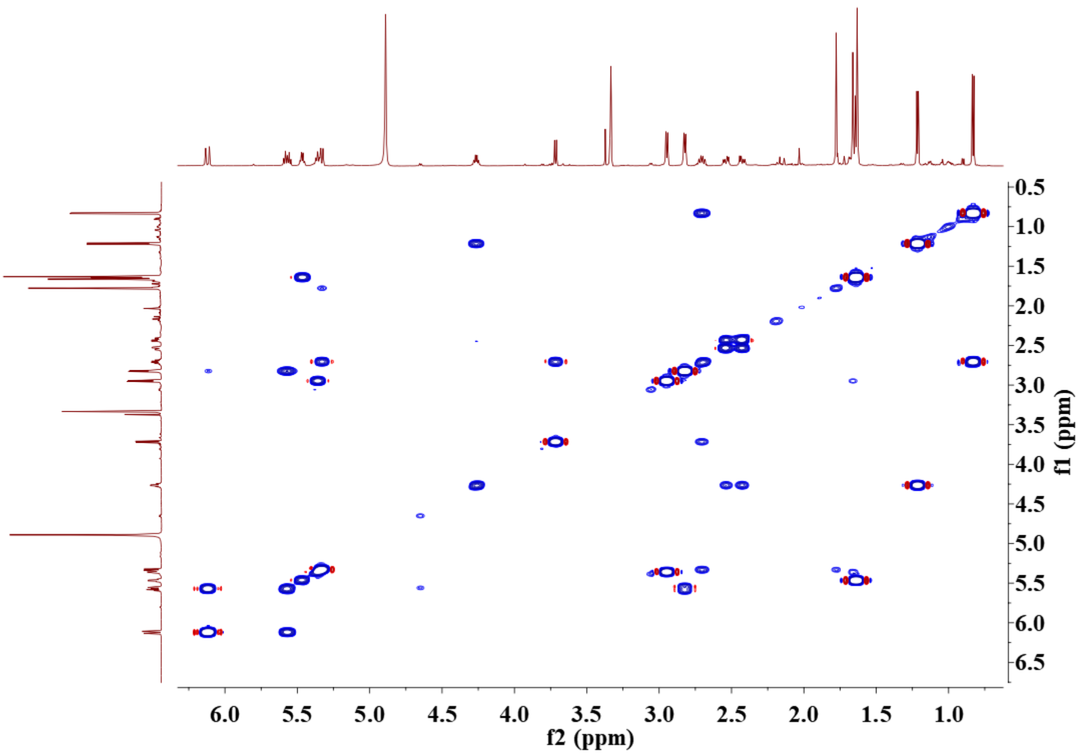

Figure S4. COSY spectrum of compound **1** in CD_3OD (600 MHz).

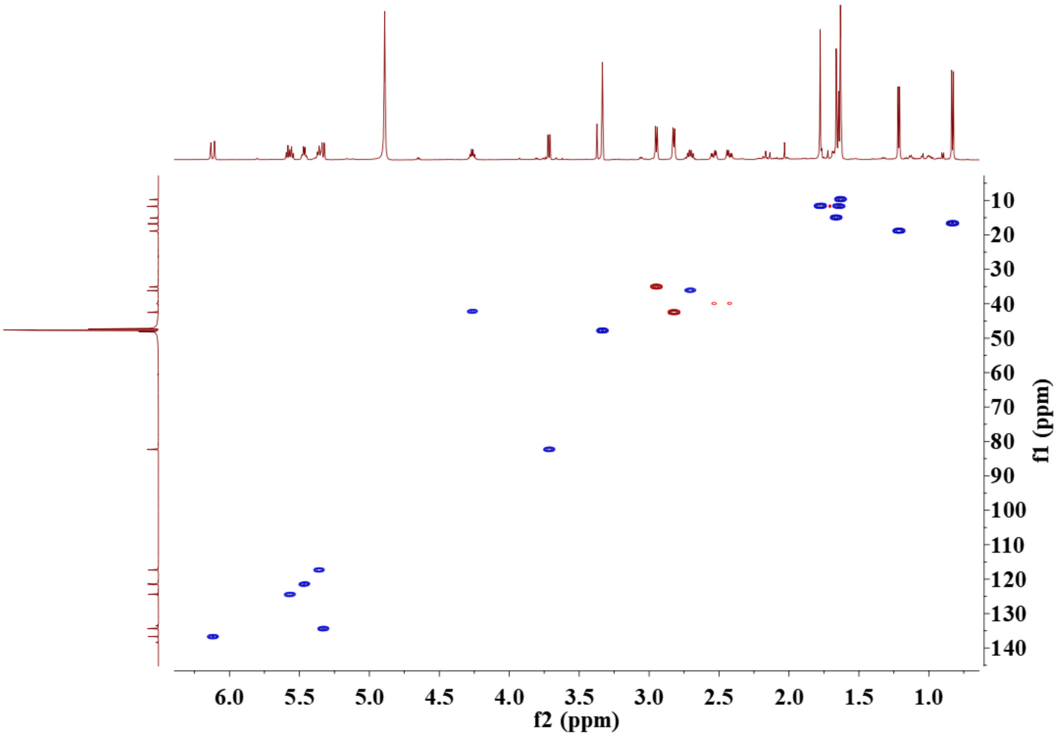

Figure S5. HSQC spectrum of compound **1** in CD_3OD (600 MHz).

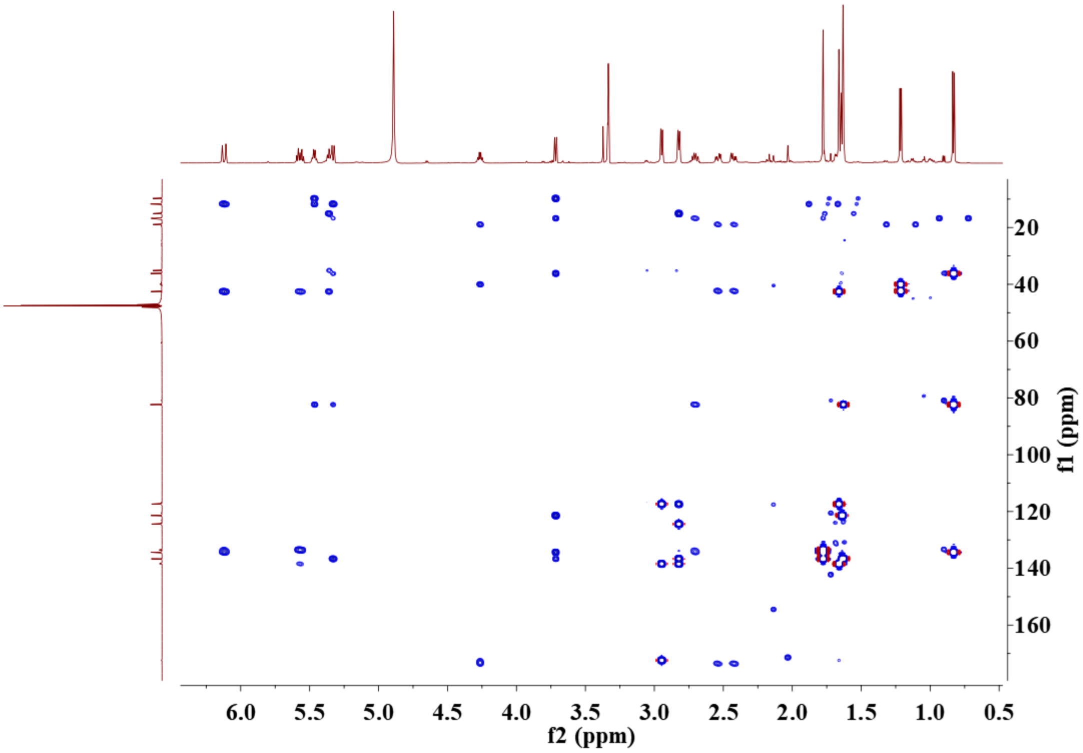

Figure S6. HMBC spectrum of compound **1** in CD_3OD (600 MHz).

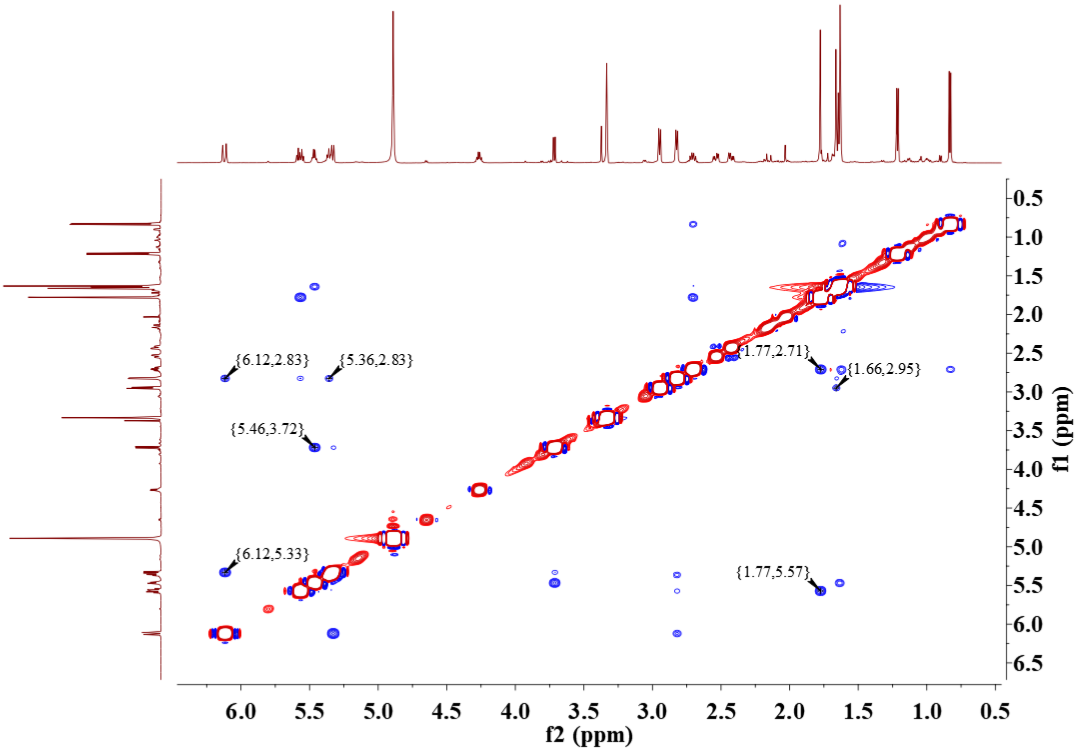

Figure S7. NOESY spectrum of compound **1** in CD3OD (600 MHz).

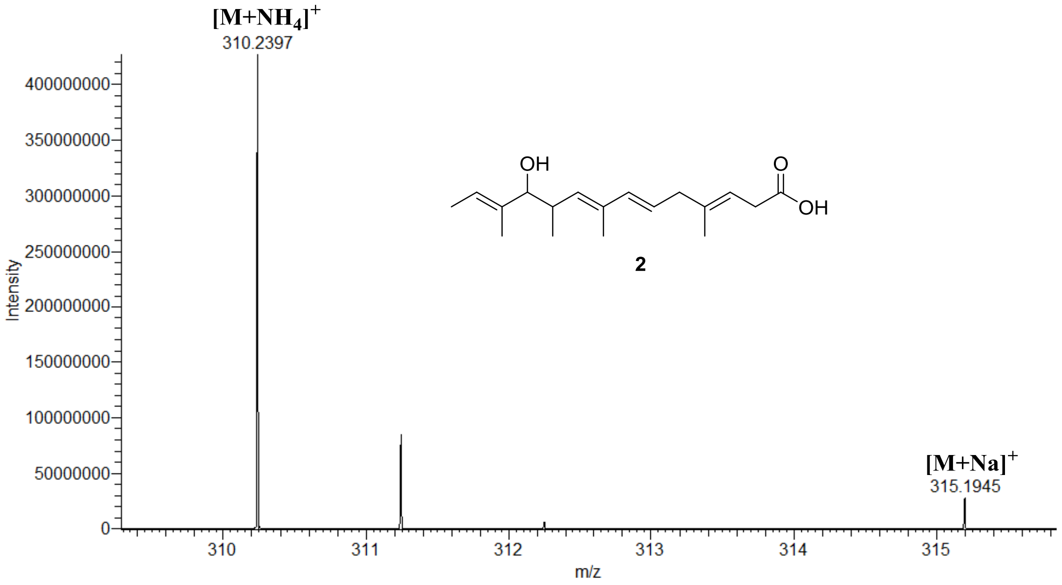

Figure S8. HR-ESIMS spectrum of compound **2**.

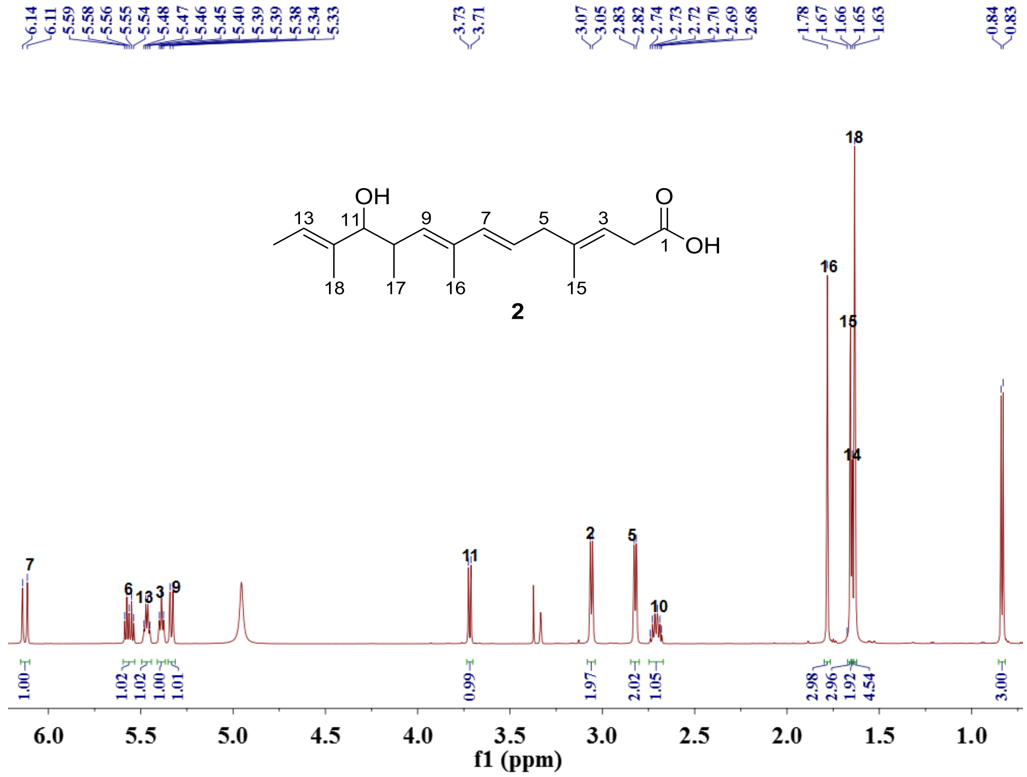

Figure S9. ^{1}H NMR spectrum of compound **2** in CD_3OD (600 MHz).

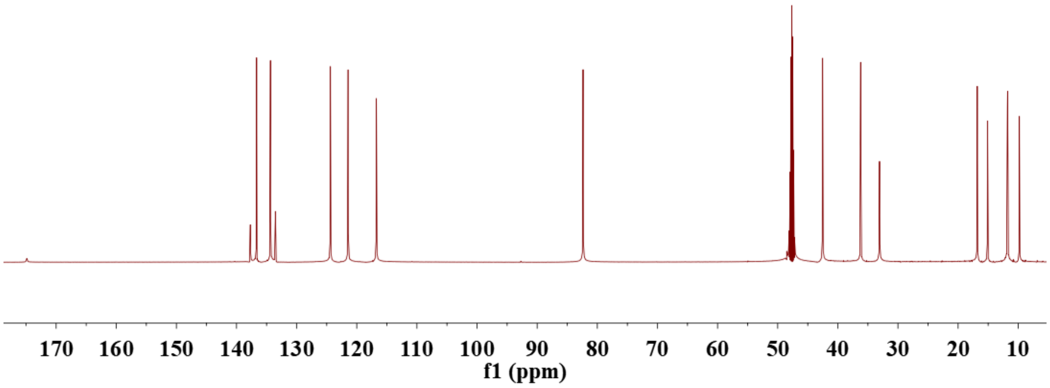

Figure S10. ^{13}C NMR spectrum of compound **2** in CD_3OD (600 MHz).

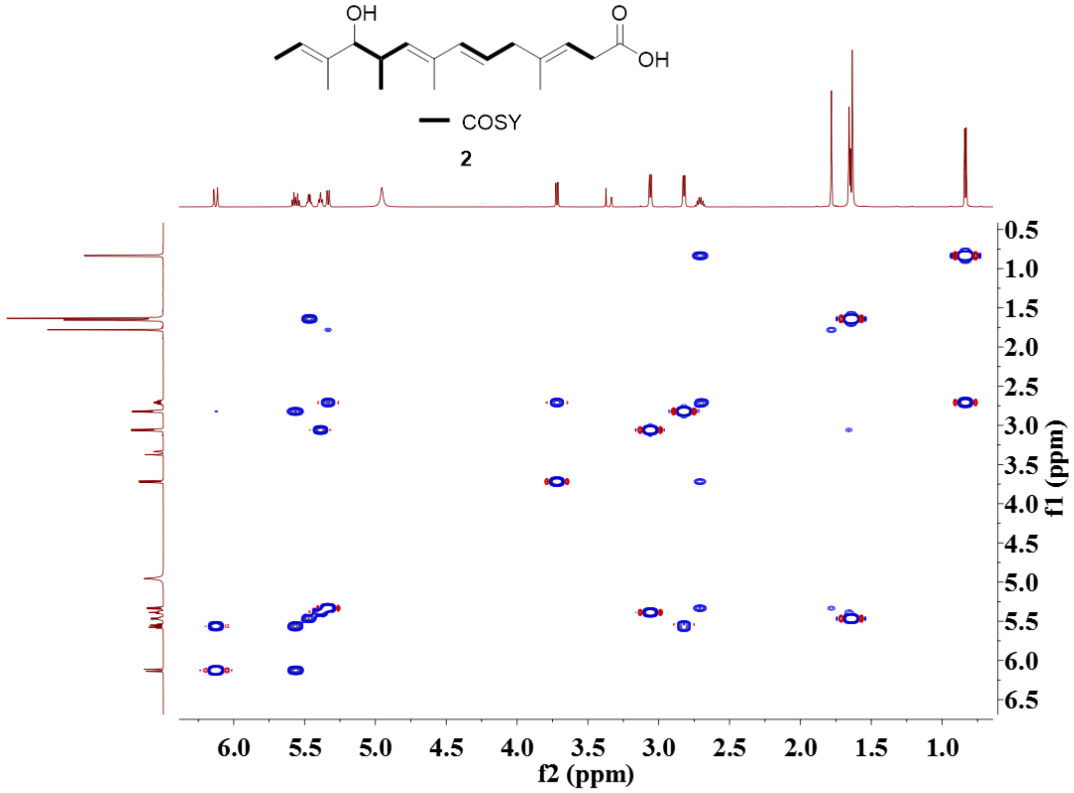

Figure S11. COSY spectrum of compound **2** in CD_3OD (600 MHz).

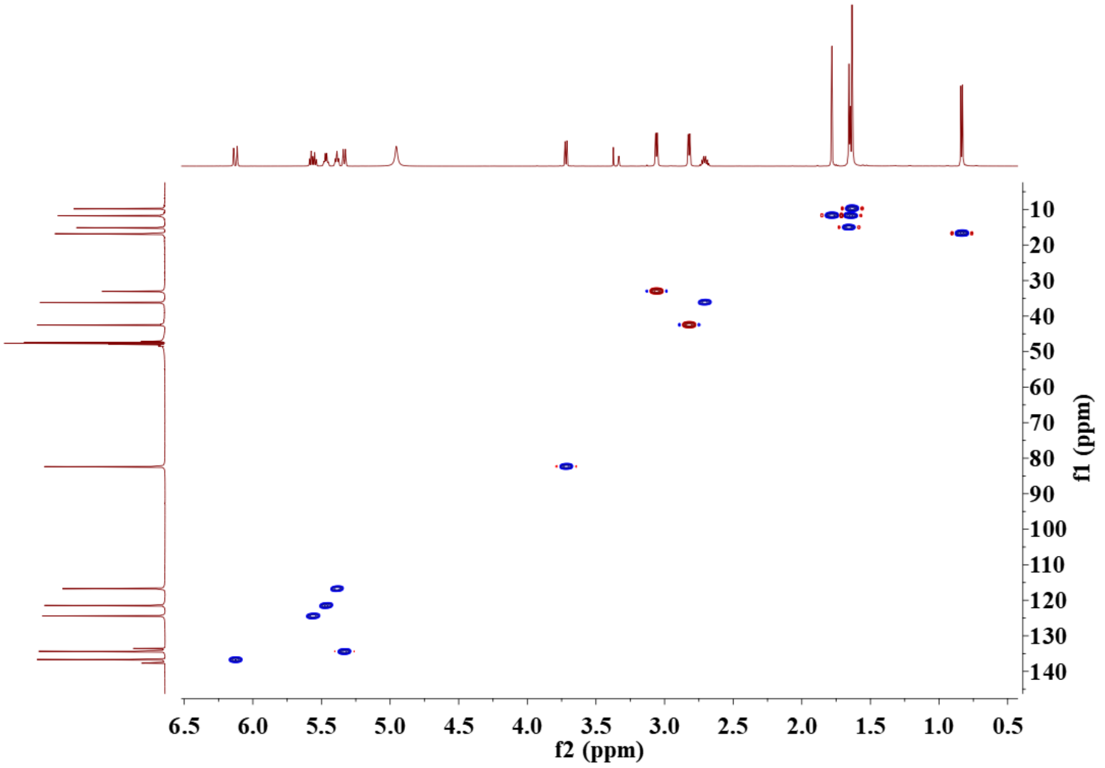

Figure S12. HSQC spectrum of compound **2** in CD_3OD (600 MHz).

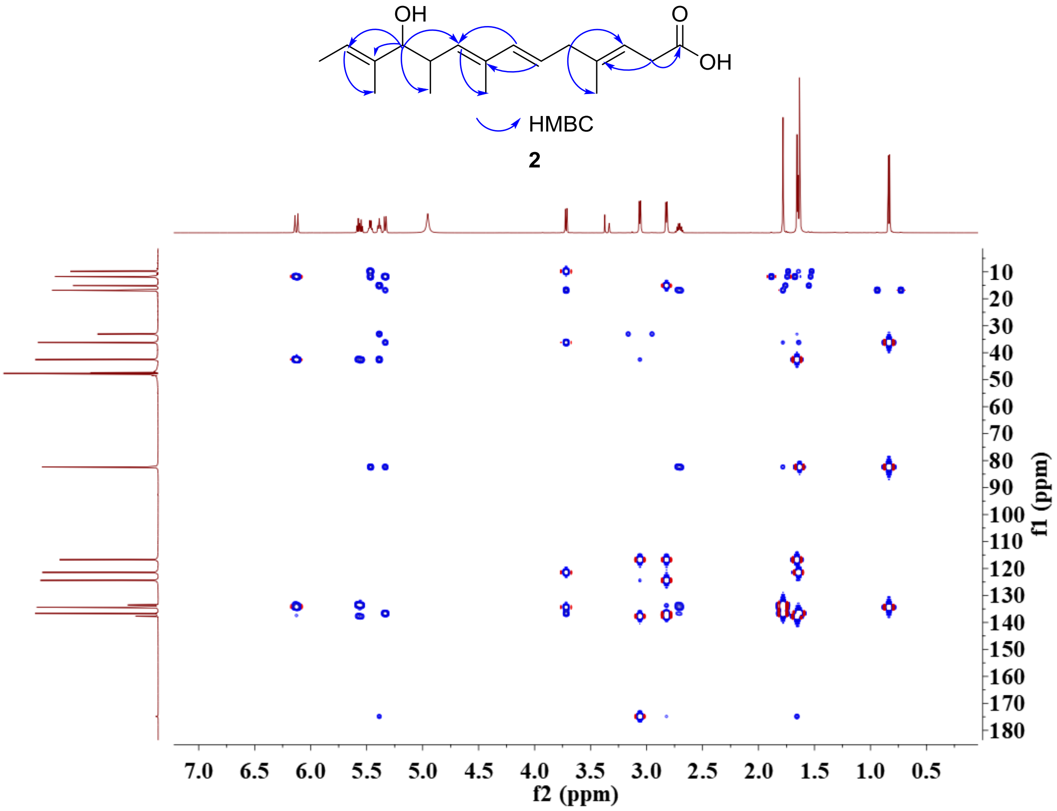

Figure S13. HMBC spectrum of compound **2** in CD_3OD (600 MHz).

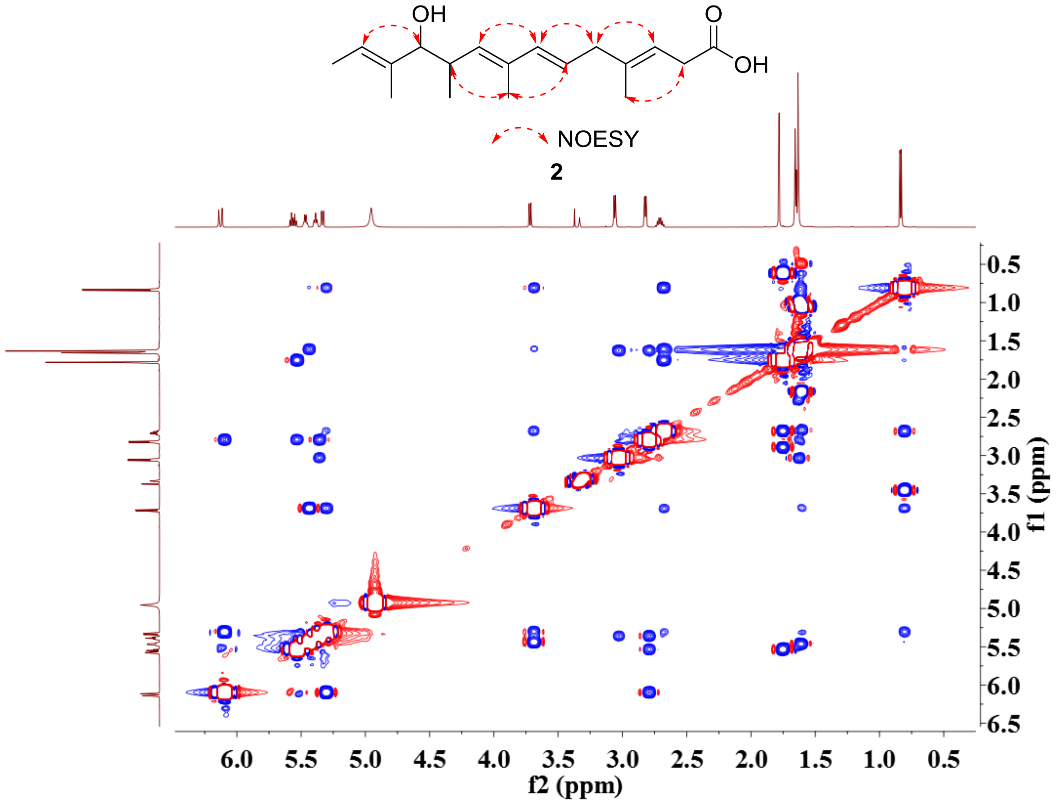

Figure S14. NOESY spectrum of compound **2** in CD_3OD (600 MHz).

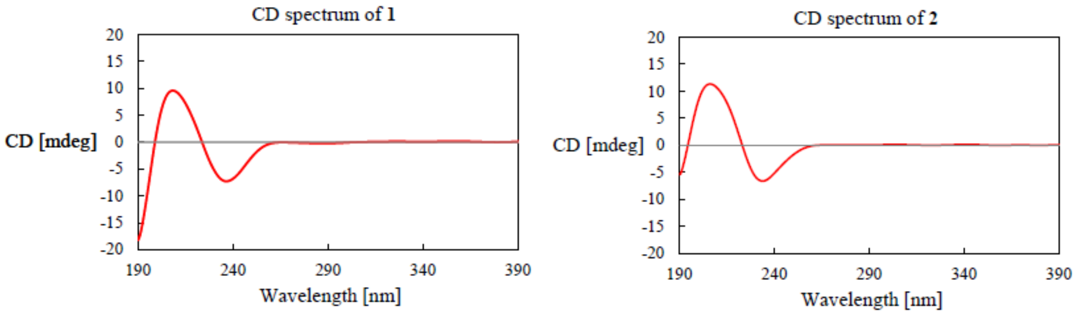

Figure S14. Experimental ECD spectra of compounds **1** and **2**.

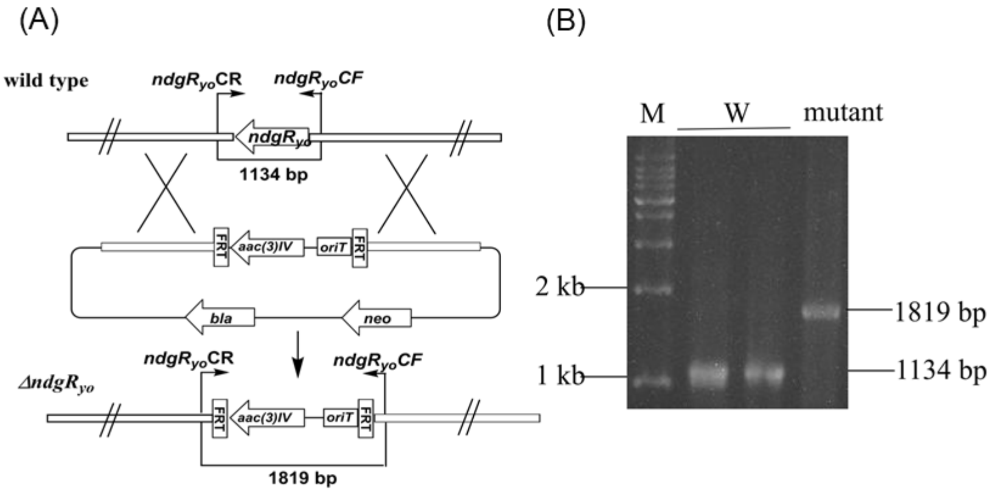

Figure S15. Inactivation of *ndgR*$_{yo}$ (A): Construction of the *ΔndgR*$_{yo}$ gene inactivation mutant. (B): PCR confirmation of the double-crossover mutant. M: 1 kb DNA marker; W: wild-type strain; Mutant: *ΔndgR*$_{yo}$ mutant.

Table S1. The primer pairs used for cosmid library screening.

cosmid	Primer pairs used for cosmid library screening (5'-3')
pWLI551	pWLI551SF:ACTCGACAAGGCTGCTCTGGT<br>pWLI551SR:GCACCGTGTCCCGCAATC

Table S2. Bacteria and plasmids used in this study.

Strains or plasmids	Description	Reference or source
Strains		
E. coli Top10	Host strain of cosmid vector SuperCos1	Invitrogen
E. coli DH5*a*	Host strain for general cloning	Stratagene
E. coli ET12567/pUZ8002	Host strain for conjugation	[1]
E. coli BW25113/pIJ790	Host strain for PCR-targeting	[2]
Streptomyces youssoufiensis OUC6819	wild type strain, Reedsmycins producer	[3]
$\Delta ndgR_{yo}$	$ndgR_{yo}$ inactivation mutant of *S. youssoufiensis* OUC6819	This study
Plasmids		
SuperCosI	Amp^R, Kan^R, cosmid vector	Stratagene
pIJ773	Apr^R, source of *acc(3)IV-oriT* cassette	[4]
pIJ790	Cm^R, λ RED recombination plasmid	[4]
pWLI551	cosmid harboring $ndgR_{yo}$ gene from *S. youssoufiensis* OUC6819	This study
pWLI552	pWLI551 derivative where $ndgR_{yo}$ was replaced with *acc(3)IV-oriT* cassette	This study

Table S3. The primer pairs used for PCR-targeted mutagenesis[a].

gene	Primer pairs used for inactivation (5'-3')
$ndgR_{yo}$	$ndgR_{yo}$MF:AGACGCGAGTATCGTTGCATGGACAACTCTAGCGGCGTGattccggggatccgtcgacc<br>$ndgR_{yo}$MR:CACGGGGCACGGGGCACGGTGGGCGTCGGGGGTGTTTCAtgtaggctggagctgcttc

[a]Underlined letters represent nucleotides homologous to the DNA regions internal to target genes

Table S5. The primer pairs used for PCR confirmation of the mutant.

gene	Primer pairs designed to verify the mutant strains (5'-3')	Fragment Replaced	Length of desired PCR fragments	
			Wild-type	Mutant
$ndgR_{yo}$	$ndgR_{yo}$CF: CGTCCCATCGCTGTCCCTC<br>$ndgR_{yo}$CR:GCCGTGGCGTAAAAGACCAA	699 bp	1134 bp	1819 bp

Biological assays

The antibacterial activity of **1** and **2** were assayed by agar diffusion method against multi-drug resistant (MDR) strains of *Enterococcus faecalis* CCARM 5172, *Enterococcus faecium* CCARM 5203, *Escherichia coli* CCARM 1009, *Salmonella typhimurium* CCARM 8250 and *Staphylococcus aureus* CCARM 3090 (Culture Collection of Antimicrobial Resistant Microbes, Seoul Women's University of Korea). The MDR strains was seeded in LB medium and then incubated at 37 ℃ for 20 h. After dilution with LB to 10^8 cfu/mL, 25 μL of cell suspension was mixed with 25 mL LB medium for each plate. Subsequently, 10 μL of compound solution (3 mg/mL) were added to the plate wells, and the inhibition zones were observed after incubation at 37 ℃ for 20 h [5].

Viabilities of human colon (HT-29) and human breast (MCF-7) cell lines were measured by sulphorhodamine (SRB) assay. Briefly, logarithmically growing cells were trypsinized from culture dishes and placed into 96- well plate. After incubation at 37 ℃ for 24 h, the compounds were added with varying concentrations. Finally, the SRB was used to stain cells, and the optical density (OD) at 540 nm was measured by a multi-detection microplate reader [6]. The 50% inhibitory concentration (IC_{50}) was determined by using a program GraphPad Prism 5.

References

1. Datsenko KA, Wanner BL. 2000. One-step inactivation of chromosomal genes in *Escherichia coli* K-12 using PCR products. *Proc. Natl Acad Sci*. 97: 6640-6645.
2. MacNeil DJ, Gewain KM, Ruby CL, Dezeny G, Gibbons PH, MacNeil T. 1992. Analysis of *Streptomyces avermitilis* genes required for avermectin biosynthesis utilizing a novel integration vector. *Gene*. 111: 61-68.
3. Che Q, Li T, Liu X, Yao T, Li J, Gu Q, Li D, Li W, Zhu T. 2015. Genome scanning inspired isolation of reedsmycins A-F, polyene-polyol macrolides from *Streptomyces* sp. CHQ-64. *RSC Adv*. 5: 22777-22782.
4. Gust B, Challis G L, Fowler K, Kieser T, Chater KF. 2003. PCR-targeted *Streptomyces* gene replacement identifies a protein domain needed for biosynthesis of the sesquiterpene soil odor geosmin. *Proc Natl Acad Sci*. 100: 1541-1546.
5. Huang H, Hou L, Li H, Qiu Y, Ju J, Li W. 2016. Activation of a plasmid-situated type Ⅲ PKS gene cluster by deletion of a *wbl* gene in deepsea-derived *Streptomyces somaliensis* SCSIO ZH66. *Microb Cell Fact*. 15: 116.
6. Rohit C, Mujahid K, Ninad S, Nitin M. 2016. A review: SRB assay for screening anticancer activity of herbal drugs (in-vitro). *Int Ayurvedic Med J*. 4: 66-70.
